# AICAR Protects against High Palmitate/High Insulin-Induced Intramyocellular Lipid Accumulation and Insulin Resistance in HL-1 Cardiac Cells by Inducing PPAR-Target Gene Expression

**DOI:** 10.1155/2015/785783

**Published:** 2015-11-16

**Authors:** Ricardo Rodríguez-Calvo, Manuel Vázquez-Carrera, Luis Masana, Dietbert Neumann

**Affiliations:** ^1^Department of Molecular Genetics, CARIM School for Cardiovascular Diseases, Faculty of Health, Medicine and Life Sciences, Maastricht University, 6200 MD Maastricht, Netherlands; ^2^Department of Pharmacology and Therapeutic Chemistry, Biomedicine Institute of University of Barcelona (IBUB), Pediatric Research Institute-Sant Joan de Déu Hospital and Spanish Biomedical Research Centre in Diabetes and Associated Metabolic Disorders (CIBERDEM), Faculty of Pharmacy, University of Barcelona, Diagonal 643, 08028 Barcelona, Spain; ^3^Vascular Medicine and Metabolism Unit, Research Unit on Lipids and Atherosclerosis, “Sant Joan” University Hospital, Pere Virgili Health Research Institute (IISPV) and Spanish Biomedical Research Centre in Diabetes and Associated Metabolic Disorders (CIBERDEM), Faculty of Medicine and Health Sciences, Rovira i Virgili University, Sant Llorenç 21, 4301 Reus, Spain

## Abstract

Here we studied the impact of 5-aminoimidazole-4-carboxamide riboside (AICAR), a well-known AMPK activator, on cardiac metabolic adaptation. AMPK activation by AICAR was confirmed by increased phospho-Thr^172^-AMPK and phospho-Ser^79^-ACC protein levels in HL-1 cardiomyocytes. Then, cells were exposed to AICAR stimulation for 24 h in the presence or absence of the AMPK inhibitor Compound C, and the mRNA levels of the three PPARs were analyzed by real-time RT-PCR. Treatment with AICAR induced gene expression of all three PPARs, but only the* Ppara* and* Pparg* regulation were dependent on AMPK. Next, we exposed HL-1 cells to high palmitate/high insulin (HP/HI) conditions either in presence or in absence of AICAR, and we evaluated the expression of selected PPAR-targets genes. HP/HI induced insulin resistance and lipid storage was accompanied by increased* Cd36*,* Acot1*, and* Ucp3* mRNA levels. AICAR treatment induced the expression of* Acadvl *and* Glut4*, which correlated to prevention of the HP/HI-induced intramyocellular lipid build-up, and attenuation of the HP/HI-induced impairment of glucose uptake. These data support the hypothesis that AICAR contributes to cardiac metabolic adaptation* via* regulation of transcriptional mechanisms.

## 1. Introduction

The heart is a metabolically highly active organ requiring continuous supply with energy to maintain proper cardiac function. It is estimated that heart consumes the equivalent of 3.5 to 5 kg of ATP every day in order to pump sufficient blood through the human body [1]. Since cardiac ATP quantity is only sufficient to sustain the cardiac function for a few seconds [1], it needs to produce energy constantly from an external supply of fuel. In a healthy heart, these energy requirements are mainly covered by fatty acids (70%) [2] and to a lesser extent by glucose (20%), and the remainder by lactate and ketone bodies [3, 4].

AMP-activated protein kinase (AMPK) is a serine/threonine kinase sensitive to cellular energy challenges. Once AMPK is activated by AMP [5, 6], it activates catabolic processes such as glycolysis and fatty acid oxidation to produce ATP and inhibits ATP-consuming processes such as protein synthesis [7, 8]. One of the main mechanisms by which AMPK contributes to restore the metabolic status is through inhibition of acetyl-CoA carboxylase (ACC) and the subsequent reduction of malonyl-CoA, an allosteric inhibitor of the carnitine palmitoyltransferase 1 (CPT-1). CPT-1 is the rate-limiting transporter controlling the delivery of fatty acids to mitochondria. Thus, AMPK acutely regulates the rate of fatty acid oxidation through ACC inhibition [9]. In addition, AMPK is also able to regulate the long-term metabolic response by modulating the activity of transcription factors, such as the peroxisome proliferator-activated receptors (PPAR) [10–14]. Thereby AMPK controls the transcription of a large number of genes involved in the regulation of both glucose and fatty acids metabolism at cellular level. The PPAR family is composed of three members, PPAR*α*, PPAR*β*/*δ*, and PPAR*γ* (NR1C1, NR1C2, and NR1C3, resp., according to the unified nomenclature system for nuclear receptors), which show different physiological roles and a tissue-specific expression pattern. PPAR*α* and PPAR*β*/*δ* are highly expressed in metabolically active tissues, such as heart, exerting functions on fatty acid uptake, activation, and *β*-oxidation [15]. It has further been proposed that the lack of one of them can be compensated by the other [15, 16], although such compensatory effect has been debated [17]. PPAR*γ* is highly expressed in white adipose tissue and immune cells, taking part in adipocyte differentiation and the regulation of glucose metabolism. Although PPAR*γ* is barely expressed in the heart, several studies have attributed to it a relevant role in the regulation of cardiac metabolism [18, 19]. PPARs are physiologically activated by long chain fatty acids and eicosanoid products, acting as ligand-dependent transcription factors. Once activated, PPARs form heterodimers with the 9-cis-retinoic acid receptor (RXR; NR2B). Then, PPAR heterodimers translocate into the nucleus and bind to specific sites in the promoter region of their targets genes, composed of an imperfect direct repeat of the hexameric sequence AGGTCA spaced by one nucleotide (DR-1), termed peroxisome proliferator response element (PPRE).

It has been previously shown that AMPK takes part in the regulation of the energy homeostasis in several tissues. At cardiac level, short-term AMPK activation protects cardiomyocytes against insulin resistance by restoring glucose uptake through mechanisms other than regulation of gene transcription [20, 21]. However, the AMPK effects that are mediated by PPARs in cardiac cells are not fully characterized as yet. In this work, we show that 5-aminoimidazole-4-carboxamide riboside (AICAR), a well-known AMPK activator [22], controls the expression of PPAR-target genes in HL-1 cardiomyocytes, counters the intramyocellular lipid accumulation induced by high palmitate/high insulin (HP/HI), and prevents the development of insulin resistance in these cells.

## 2. Methods

### 2.1. Reagents

2-Deoxy-D-[^3^H]-deoxyglucose was obtained from GE Healthcare. Palmitate, insulin, and bovine serum albumin (BSA) were purchased from Sigma.

### 2.2. Cell Culture

HL-1 atrial cardiomyocytes were kindly provided by W. Claycomb (Louisiana State University, New Orleans, LA, USA) and cultured in Claycomb medium (supplemented with 10% FCS, 0.1 mmol/L noradrenaline [norepinephrine], 2 mmol/L L-glutamine, 10 U/mL penicillin, and 100 *μ*g/mL streptomycin) at 37°C and 5% CO_2_. Cells were seeded in multiwell plates and were serum-deprived in DMEM (supplemented with 2 mM L-glutamine, 100 *μ*M nonessential amino acids (NEAA), 100 U/mL penicillin, and 100 *μ*g/mL streptomycin) for 24 h and were then stimulated with AICAR (0.5 mM) for 1 or 24 h in the presence or absence of Compound C (5 *μ*M). In another set of experiments, cells stimulated or not with AICAR (0.5 mM, 24 h) were challenged with high palmitate (500 *μ*M, palmitate : BSA 3 : 1)/high insulin (100 nM) (HP/HI) for the last 16 h.

### 2.3. Preparation of the Palmitate-BSA Complex

Palmitate was dissolved in ethanol in a glass container and then mixed with a water solution containing KOH 1 N. Then, ethanol was evaporated under nitrogen at 45°C, and palmitate solution was slowly added dropwise to a shaking prewarmed 2% BSA-containing solution to reach the final concentration desired.

### 2.4. Immunoblotting

Whole cellular extracts were obtained using RIPA buffer (50 mM Tris-HCl, 150 mM NaCl, 1% Igepal, 0.5% sodium deoxycholate, and 0.1% SDS containing proteases and phosphatases inhibitors). Protein concentration was measured by the BCA protein assay and equal amounts were resolved by SDS-PAGE and transferred to Immobilon polyvinylidene difluoride (PVDF) membranes. Western blots analysis was performed using antibodies against phospho-Thr^172^-AMPK, total AMPK, phospho-Ser^473^-AKT, total AKT (Cell Signaling), phospho-Ser^79^-ACC, and total ACC (Upstate). Detection was performed using the appropriate horseradish peroxidase-labelled IgG and the Chemiluminescent Peroxidase Substrate-1 (Sigma). The size of detected proteins was estimated using protein molecular-mass standards (Thermo Scientific, Waltham, MA, USA). Western blot images were analyzed with a Molecular Imager (ChemiDoc XRS, BioRad) and quantified with Quantity One (BioRad).

### 2.5. RNA Preparation and Quantitative Real-Time RT-PCR Analysis

Levels of mRNA were assessed by the real-time reverse transcription-polymerase chain reaction (real-time RT-PCR). Total RNA was isolated using the TRI Reagent (Sigma, Saint Louis, USA) according to the manufacturer's recommendations. RNA integrity was determined by electrophoresis in agarose gel. Total RNA (1 *μ*g) was reverse-transcribed using the iScript cDNA Synthesis Kit (BioRad). Levels of mRNA were assessed by real-time PCR on an ABI PRISM 7900 sequence detector (Applied Biosystems). Primers for SYBR Green real-time PCR analysis of peroxisome proliferator-activated receptor *α* (*Ppara*) (5′-ATGATGGGAGAAGATAAAATCAAGTTC-3′ and 5′-CGGCTTCTACGGATCGTTTC-3′),* Ppard* (5′-TGTGCAGCGGTGTGGGTAT-3′ and 5′-GTCATAGCTCTGCCACCATCTG-3′),* Pparg* (5′-GAAGTTCAATGCACTGGAATTAGATG-3′ and 5′-CCTCGATGGGCTTCACGTT-3′),* Cd36* (5′-GCCAAGCTATTGCGACATGA-3′ and 5′-AAAAGAATCTCAATGTCCGAGACTTT-3′), acyl-CoA thioesterase 1 (*Acot1*) (5′-GCAGCCACCCCGAGGTAAA-3′ and 5′-GCCACGGAGCCATTGATG-3′), carnitine palmitoyltransferase I (*Cpt1b*) (5′-GCCCCCTCATGGTGAACAG-3′ and 5′-TGGCGTGAACGGCATTG-3′), acyl-CoA oxidase (*Acox1*) (5′-TGTGACCCTTGGCTCTGTTCT-3′ and 5′-TGTAGTAAGATTCGTGGACCTCTG-3′), acyl-CoA dehydrogenase, very long chain (*Acadvl*) (5′-AGACGGAGGACAGGAATCGG-3′ and 5′-ACCACGGTGGCAAATTGATC-3′), uncoupling protein-3 (*Ucp3*) (5′-GGATTTGTGCCCTCCTTTCTG-3′ and 5′-CATTAAGGCCCTCTTCAGTTGCT-3′),* Gut4* (5′-GCTTTGTGGCCTTCTTTGAG-3′ and 5′-CAGGAGGACGGCAAATAGAA-3′), and pyruvate dehydrogenase kinase (*Pdk4*) (5′-GCATTTCTACTCGGATGCTCATG-3′ and 5′-CCCAAGCCACATTGG-3′) were used.* Cyclophilin A* (5′-TTCCTCCTTTCACAGAATTATTCCA-3′ and 5′-CCGCCAGTGCCATTATGG-3′) was used as endogenous control.

### 2.6. Oil-Red-O Staining

Lipid content was measured in HL-1 cells challenged with HP/HI in the presence or absence of AICAR. Cells were fixed in ice-cold 4% paraformaldehyde for 15 min and stained with fresh Oil-Red-O (Sigma) solution for 30 min. Nuclei were counterstained with Haematoxylin and cells were mounted with Faramount mounting medium (Dako) after extensive washing. Pictures were taken at 40x magnification with a Nikon digital camera DMX1200 and ACT-1 v2.63 software from Nikon Corporation. The lipid content was quantified by Image J software from five random fields of three different experiments.

### 2.7. Measurement of 2-Deoxy-D-[^3^H]-glucose Uptake

2-Deoxy-D-[^3^H]-glucose uptake was measured as previously described [23] in HL-1 cells stimulated with HP/HI in the presence or absence of AICAR. Briefly, cells were washed with uptake-buffer (117 mM NaCl, 2.6 mM KCl, 1.2 mM KH_2_PO_4_, 1.2 mM MgSO_4_, 10 mM NaHCO_3_, 10 mM HEPES, and 1 mM CaCl_2_) containing 4.6 mg/mL BSA and challenged with 200 nM insulin for 30 min. Subsequently, deoxy-D-glucose was added to final concentration of 4 *μ*M with tracer amounts of 2-deoxy-D-[^3^H]-glucose (~2.17 *μ*Ci). After 10 min, uptake was stopped with ice-cold stop-solution (uptake-buffer containing 1 mg/mL BSA, 0.2 mM phloretin, and 0.1% of DMSO). Then, cells were lysed with 1 M NaOH, and incorporated glucose was measured by scintillation counting of ^3^H in a *β*-counter.

### 2.8. Statistical Analyses

Results are expressed as mean ± SD. Significant differences were established by Student's *t*-test using the GraphPad Instat programme (GraphPad Software V2.03). Differences were considered significant at *P* < 0.05.

## 3. Results

### 3.1. AICAR Induces PPAR Expression in HL-1 Cardiac Cells

**Figure 1:**
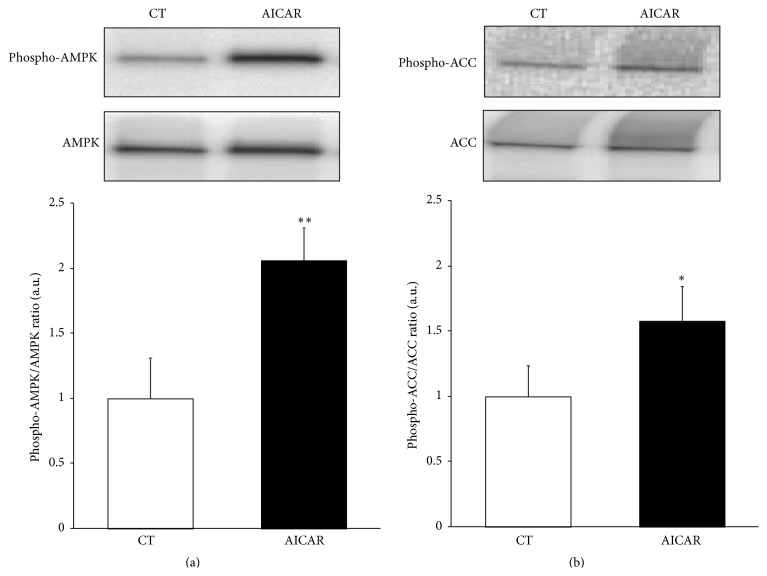
AICAR induces AMPK activation in HL-1 cardiac cells. HL-1 cells were stimulated with AICAR for 1 h and the AMPK activation was confirmed by the protein levels of phospho-Thr^172^-AMPK (a) and phospho-Ser^79^-ACC (b). Quantifications show the ratio between phosphorylated and total forms of each protein. Data are expressed as mean ± SD of 3 different experiments. (^*∗*^ *P* < 0.05, ^*∗∗*^ *P* < 0.01* versus* control nonstimulated cells).

**Figure 2:**
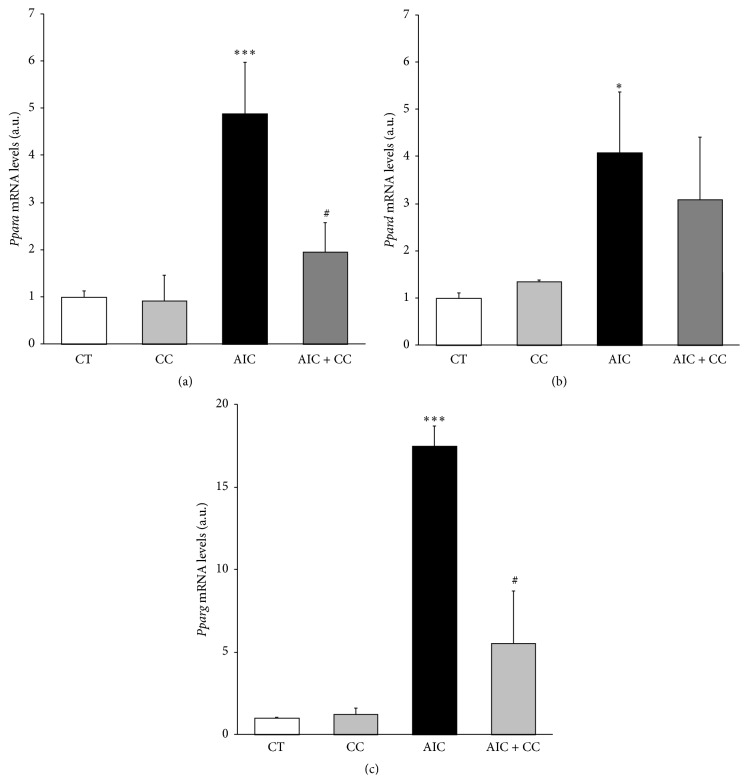
AICAR upregulates the mRNA levels of the three PPARs in HL-1 cardiomyocytes. Analysis of the mRNA levels of* Ppara* (a),* Ppard* (b), and* Pparg* (c) by real-time RT-PCR in HL-1 cells stimulated by AICAR for 24 h in the presence or absence of Compound C. Data are normalized by the* Cyclophilin A* mRNA levels and expressed as mean ± SD of 4 different experiments. (^*∗*^ *P* < 0.05, ^*∗∗∗*^ *P* < 0.001* versus* control nonstimulated cells; ^#^ *P* < 0.05* versus* AICAR-treated cells).

AICAR-induced AMPK activation in HL-1 cardiac cells has been previously reported by others [24]. To confirm this in our hands, HL-1 cells were treated with AICAR for 1 h and AMPK phosphorylation was analyzed. AICAR treatment induced AMPK phosphorylation in Thr 172 (~2-fold, *P* < 0.01) (Figure 1(a)) as well as phosphorylation in Ser 79 of its well-known target ACC (~1.6-fold, *P* < 0.05) (Figure 1(b)), confirming that AICAR stimulation activates AMPK in HL-1 cardiomyocytes. Because AMPK is able to drive the long-term metabolic adaptation through PPAR regulation [10–14], we explored the effect of AICAR stimulation for 24 h on PPAR expression. Treatment with AICAR strongly induced the* Ppara* (~4.9-fold, *P* < 0.001) (Figure 2(a)),* Ppard* (~4.1-fold, *P* < 0.05) (Figure 2(b)), and* Pparg* (~17.5-fold, *P* < 0.001) (Figure 2(c)) mRNA levels. However, while AICAR-induced* Ppara* and* Pparg* expression was attenuated in the presence of the AMPK inhibitor Compound C (Figures 2(a) and 2(c)), this drug was unable to prevent the AICAR-induced* Ppard* upregulation (Figure 2(b)).

### 3.2. AICAR Regulates the Expression of PPAR-Target Genes in HP/HI-Stimulated Cells

**Figure 3:**
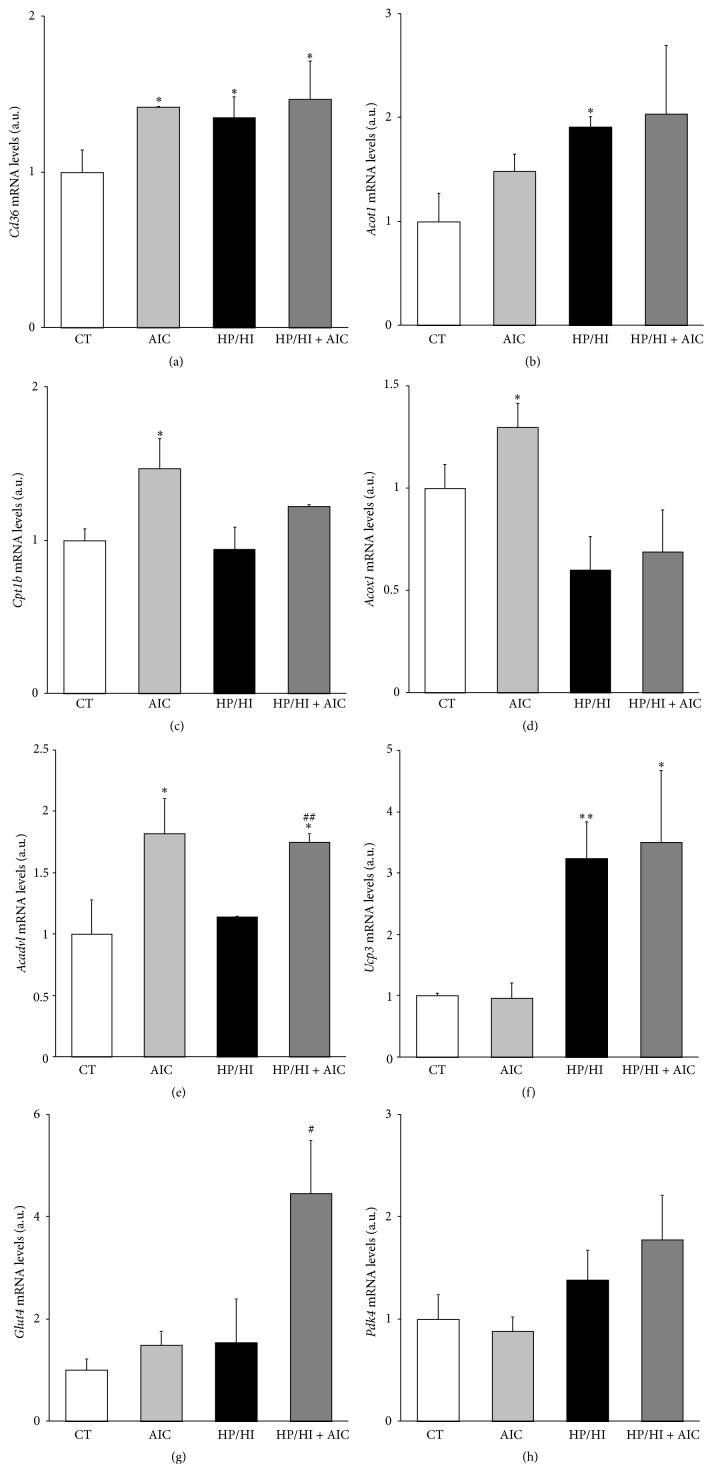
Treatment with AICAR regulates the expression of PPAR-target genes. Analysis of the mRNA levels of* Cd36* (a),* Acot1* (b),* Cpt-1b* (c),* Acox1* (d),* Acadvl* (e),* Ucp3* (f),* Glut4* (f), and* Pdk4* (h) by real-time RT-PCR in HL-1 cells stimulated with HP/HI for 16 h in the presence or absence of AICAR (24 h). Data are normalized by the* Cyclophilin A* mRNA levels and expressed as mean ± SD of 4 different experiments. (^*∗*^ *P* < 0.05, ^*∗∗*^ *P* < 0.01* versus* control nonstimulated cells; ^#^ *P* < 0.05, ^##^ *P* < 0.01* versus* HP/HI-stimulated cells).

Next, HL-1 cardiomyocytes were challenged with HP/HI to render the cells insulin resistant, in the presence or absence of AICAR, and the expression of selected PPAR-target genes involved in glucose and fatty acid metabolism was analyzed by real-time PCR. HP/HI stimulation induced the expression of the fatty acid transporter* Cd36* (~1.4-fold; *P* < 0.05* versus* CT) (Figure 3(a)),* Acot1* (~1.9-fold; *P* < 0.05* versus* CT) (Figure 3(b)), and* Ucp3* (~3.2-fold; *P* < 0.01* versus* CT) (Figure 3(f)). Nevertheless, HP/HI stimulation did not alter the expression of other genes involved in fatty acid and glucose metabolism, such as* Cpt1b* (Figure 3(c)),* Acox1* (Figure 3(d)),* Acadvl* (Figure 3(e)),* Glut4* (Figure 3(g)), and* Pdk4* (Figure 3(h)). Treatment with AICAR induced the expression of key genes involved in fatty acid metabolism, such as* Cd36* (~1.4-fold; *P* < 0.05* versus* CT) (Figure 3(a)),* Cpt1b* (~1.5-fold; *P* < 0.05* versus* CT) (Figure 3(c)),* Acox1* (~1.3-fold; *P* < 0.05* versus* CT) (Figure 3(d)), and* Acadvl* (~1.8-fold; *P* < 0.05* versus* CT; ~1.8-fold; *P* < 0.01* versus* HP/HI) (Figure 3(e)), and glucose transport, such as* Glut4* (~4.5-fold; *P* < 0.05* versus* HP/HI) (Figure 3(g)).

### 3.3. AICAR Prevents HP/HI-Induced Intramyocellular Lipid Accumulation

**Figure 4:**
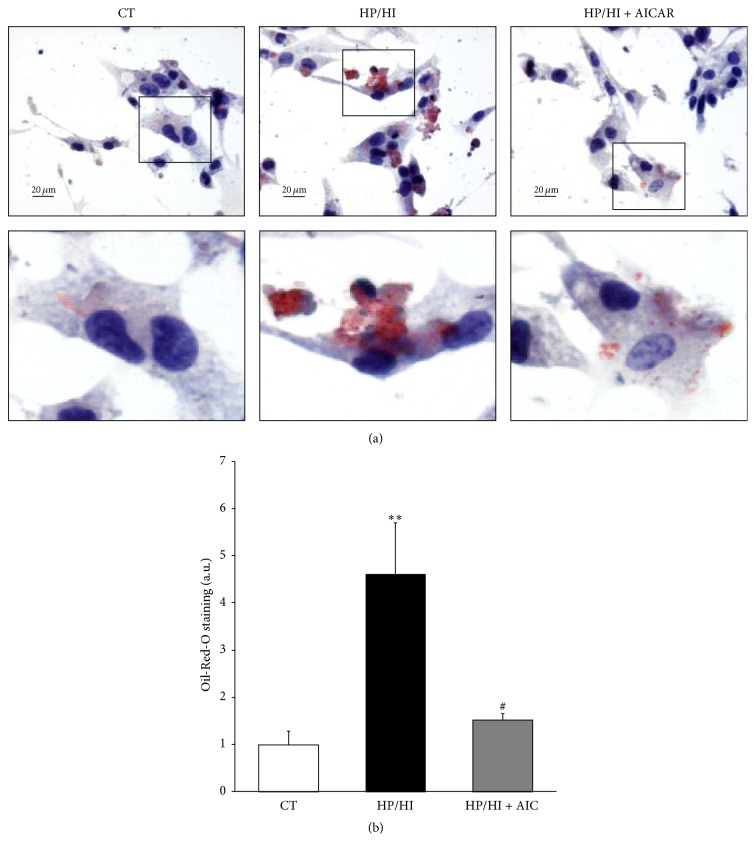
AICAR prevents the HP/HI-induced intramyocellular lipid accumulation in HL-1. Lipid content was analyzed by Oil-Red-O staining in HL-1 challenged with HP/HI for 16 h in the presence or absence of AICAR (24 h). (a) Representative microphotography showing lipid droplets in cells counterstained with Haematoxylin (bar 20 *μ*m). Squares indicate the areas shown at high magnification. (b) Quantification of stained areas relative to cell surface. Data are expressed as mean ± SD of 5 different pictures from 3 independent experiments (^*∗∗*^ *P* < 0.01* versus* control nonstimulated cells; ^#^ *P* < 0.05* versus* HP/HI-stimulated cells).

In order to explore whether AICAR-induced* Acadvl* expression was related to changes in the intramyocellular lipid content, we performed Oil-Red-O staining in HL-1 cells challenged with HP/HI. HP/HI increased the lipid accumulation (~4.6-fold; *P* < 0.01* versus* CT) compared to nonstimulated cells. However, AICAR treatment prevented the effect of HP/HI on intramyocellular lipid accumulation (Figures 4(a) and 4(b)).

### 3.4. AICAR Improves Glucose Uptake in HP/HI-Stimulated Cells

**Figure 5:**
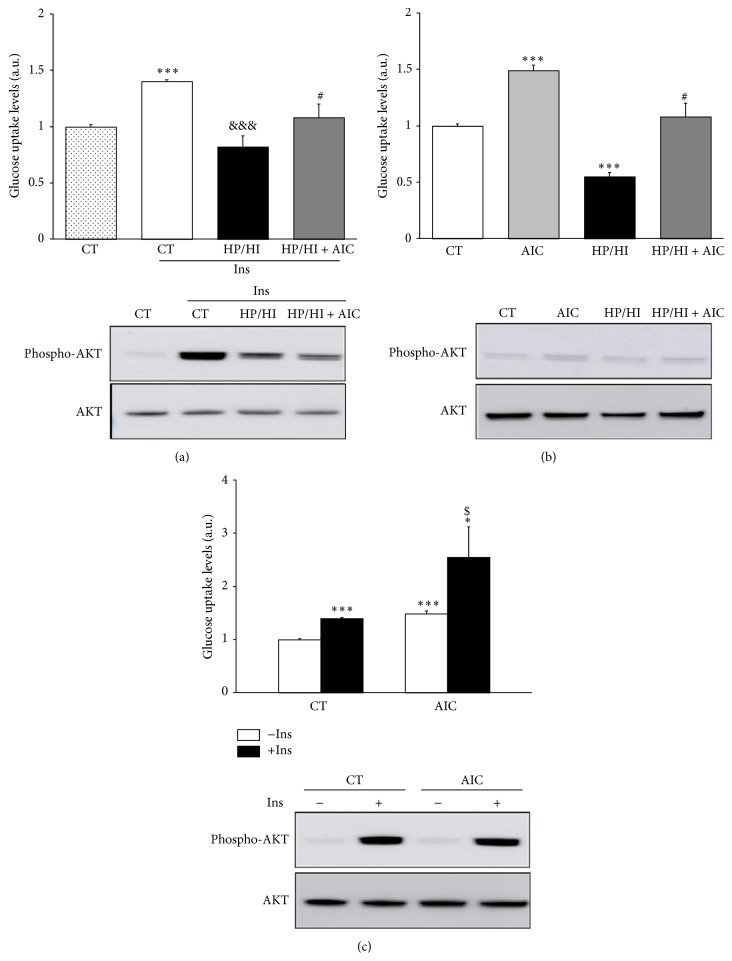
AICAR stimulation attenuates HP/HI-induced glucose uptake impairment in HL-1. HL-1 cells were stimulated with HP/HI for 16 h in the presence or absence of AICAR (24 h), and [^3^H]-deoxyglucose uptake (up) and AKT phosphorylation (down) were assessed in the presence (a) and absence (b) of insulin (200 nmol/L, 10 min). [^3^H]-deoxyglucose uptake (up) and AKT phosphorylation (down) determination in HL-1 cells stimulated with AICAR (24 h) in the presence and absence of insulin (200 nmol/L, 10 min) (c). Data are expressed as mean ± SD of 4 different experiments performed in duplicate. (^*∗*^ *P* < 0.05, ^*∗∗∗*^ *P* < 0.001* versus* control cells without insulin stimulation; ^&&&^ *P* < 0.001* versus* control cells stimulated with insulin; ^#^ *P* < 0.05* versus* HP/HI-challenged cells stimulated with insulin (a) or without insulin (b); ^$^ *P* < 0.05* versus* AICAR-treated cells stimulated with insulin).

Because GLUT4 is the main glucose transporter in HL-1 cardiomyocytes, we wonder whether the AICAR-induced* Glut4* mRNA levels for 24 h may be related to a transcriptional metabolic adaptation aimed at preparing the cell for increasing glucose uptake. Thus, we assessed the effect of AICAR treatment over insulin stimulated glucose uptake and AKT phosphorylation in HL-1 cardiomyocytes challenged with HP/HI. As shown in Figure 5(a), acute insulin stimulation induced glucose uptake (~1.4-fold; *P* < 0.001* versus* CT-Ins) and AKT phosphorylation. However, HP/HI-stimulated cells were not sensitive to insulin (−41.3%; *P* < 0.001* versus* CT + Ins). AICAR treatment prevented the HP/HI effects reducing glucose uptake (~1.3-fold; *P* < 0.05* versus* HP/HI + Ins) and AKT phosphorylation (Figure 5(a)). Because AICAR is able to stimulate glucose uptake in a non-insulin dependent way [25, 26], we explored the effect of this drug in the absence of insulin. AICAR itself induced glucose uptake (~1.5-fold; *P* < 0.001* versus* CT) and prevented HP/HI-induced glucose uptake downregulation (Figure 5(b)), with no changes in the AKT phosphorylation state. Finally, to further clarify the action of this drug on insulin-response, the additive action of insulin and AICAR was evaluated. Combination of AICAR and insulin showed a synergistic effect on glucose uptake (~1.8-fold; *P* < 0.05* versus* CT + Ins; ~1.7-fold; *P* < 0.05* versus *AICAR − Ins), but not on the AKT phosphorylation (Figure 5(c)).

## 4. Discussion

The heart is able to adapt metabolism in order to produce the energy that is needed to maintain proper function (for review see [27]). Although in adult healthy heart the energy requirements are mainly covered by fatty acids and glucose, heart is able to shift its substrate preference when facing certain physiological or pathological conditions, such as fasting, insulin resistance, or diabetes. At molecular level, these processes are accurately regulated by the “metabolic master switch” AMPK. Here, we show that AICAR, a well-known AMPK activator, induces the expression of the PPAR family of nuclear receptors and protects cardiac HL-1 cells from HP/HI-induced intramyocellular lipid accumulation and insulin resistance.

AICAR-induced changes in the PPARs protein expression and transcriptional activity have been previously reported by others in several cell types, including cardiomyocytes [10–12, 14]. In addition, the cross talk between AMPK and PPARs has been also shown [10–14]. Although the molecular mechanisms underlying AMPK-induced regulation of PPARs are not completely understood, several lines of evidence indicate changes in the PPARs mRNA and protein levels [10–12], suggesting the involvement of transcriptional mechanisms. AMPK also regulates the peroxisome proliferator-activated receptor-*γ* coactivator (PGC-1) [28], which enhances the PPARs transcriptional activity. In addition, AMPK is able to modulate the PPAR activity by phosphorylation [11]. In cardiac tissue, adiponectin induces AMPK-mediated PPAR*α* phosphorylation [29] and protects against angiotensin II-induced cardiac fibrosis through a mechanism involving the AMPK-dependent PPAR*α* activation [30]. Moreover, AICAR-induced AMPK activation prevents the PPAR*α* reduction in both* in vitro* and* in vivo* models of cardiac hypertrophy [10, 11]. Furthermore, it has been recently shown that AMPK activation by Metformin protects from oxidative stress in H9c2 cardiomyoblasts, avoiding the physical interaction between PPAR*α* and Cyclophilin D [31]. Nevertheless, although AMPK also regulates the PPAR*β*/*δ* and PPAR*γ* expression in other cell types, these regulations in cardiac cells are not completely unveiled [12, 32]. Here, we show that AICAR stimulation induced the expression of the three PPARs in HL-1 cardiomyocytes. AICAR upregulated* Ppara* and* Ppparg* mRNA levels, which was blunted by the AMPK inhibitor Compound C. Although it is rather known that Compound C has several AMPK-unrelated actions [33], Fryer et al. demonstrated that Compound C blunted the AICAR-induced AMPK activation [34], supporting that the inductions of* Ppara* and* Ppparg* by AICAR were dependent on AMPK. However, Compound C was unable to prevent the AICAR-induced* Ppard* expression, indicating the involvement of AMPK-independent mechanisms.

Since PPARs are major regulators of glucose and fatty acid metabolism at transcriptional level, we explored the effect of AICAR-induced PPAR expression on selected PPAR-target genes in HP/HI-stimulated cells. Fatty acids, such as palmitate, are natural ligands of PPARs, which in turn are able to induce the expression of some target genes after short-term exposure to fatty acids [13]. Nevertheless, such regulation is not observed in case of sustained stimulations with palmitate, probably due to reduction in the PPAR*α* and PPAR*δ* levels [13]. The aberrant PPAR regulation is closely related to cardiac lipotoxicity and the build-up of intramyocellular lipids, which contribute to metabolic disturbances related to insulin resistance and diabetic heart [13, 25, 26, 35–37]. For instance, PPAR*α* levels have been found reduced in cardiomyocytes chronically exposed to fatty acids excess [35] and in hearts from senescence-accelerated mice with enhanced ceramide levels [36]. Unlike other PPARs, PPAR*γ* is barely detectable in heart, but it is upregulated in hearts from rat models of DM [25, 26, 37], thereby contributing to the storage of intramyocellular lipid content [37]. We found that HP/HI stimulation induced the expression of* Cd36*,* Acot1*, or* Ucp3*, suggesting a transcriptional reprograming aimed at increasing the fatty acid uptake and mitochondrial uncoupling. Uncoupling mitochondrial respiratory chain from oxidative phosphorylation could be an adaptive mechanism promoting the burning of the toxic lipid stores. Actually, increased UCP3 activity in skeletal muscle has been associated with increased fatty acid oxidation rates [38]. Nevertheless, because heart needs to produce energy constantly, induction in the* Ucp3* expression has been previously reported in diabetic hearts like a hallmark of contractile dysfunction [39]. Thus, although in the early response to HP/HI PPARs could take part in the regulation of a transcriptional program aimed at increasing fatty acid utilization in insulin resistant cardiomyocytes, sustained fatty acid exposure seems to be related to a decrease in the PPAR*α* and *δ*-induced fatty acid oxidation and an increased PPAR*γ*-induced fatty acid uptake and accumulation. In our cellular model, AICAR stimulation induced the expression of* Cd36*,* Cpt1b*,* Acox1*, and* Acadvl*, compared to nonstimulated cells, without affecting the expression of other PPAR-targets. Additionally, AICAR treatment enhanced* Acadvl* and* Glut4* mRNA levels in HP/HI-challenged cells. Therefore, our data indicates that AICAR can only activate a subset of PPAR-target genes whereas additional signals are needed for other targets.

AICAR-induced changes in* Acadvl* expression correlated to the effect of this compound preventing the raise in cardiac lipid accumulation by HP/HI. The deficiency in the* Acadvl* product (very long-chain acyl-CoA dehydrogenase, VLCAD) reduces myocardial fatty acid *β*-oxidation and is associated with cardiomyopathy [40]. Because* Acadvl* catalyzes the first step of the mitochondrial *β*-oxidation of long chain and very long chain fatty acids, our data suggest that AICAR may enhance lipid mitochondrial *β*-oxidation by a previously unrecognized mechanism that is different from the established acute AMPK-dependent regulation of fatty acid metabolism by ACC phosphorylation [9]. Relevantly, long chain and very long chain fatty acids are the major components of storage triglycerides and derivatives (diacylglycerols, ceramides) and are the precursors of major lipid signalling molecules, such as prostaglandins and leukotrienes [41]. Furthermore, intramyocellular lipid accumulation activates Ser/Thr-kinase cascades, enhancing insulin resistance through impairment of both insulin stimulated glucose uptake [42] and oxidation [20]. Therefore, just reducing the intramyocellular lipid content AICAR may contribute to restoration of the insulin signalling pathway. In addition, AMPK activation by AICAR after short-term stimulation promotes GLUT4 translocation to cellular membrane* via* a non-insulin dependent mechanism [21, 43]. Interestingly, this acute AMPK regulation excludes modifications in gene transcription. Here, we show that longer exposure of HL-1 with AICAR further induced the* Glut4* mRNA levels, suggesting a transcriptional metabolic adaptation aimed at preparing the cell for increasing glucose uptake. Thus, apart from the previously reported mechanisms our data reveal that AICAR-induced* Glut4* upregulation correlated to the effect of this drug restoring the reduction of glucose uptake after HP/HI challenge, in both the presence and absence of insulin. Additionally, a synergistic effect on glucose uptake was found in cells stimulated with both insulin and AICAR, which was not observed in the AKT phosphorylation levels, suggesting that the effect of AICAR preventing downregulation of glucose uptake in HP/HI-stimulated cells is not dependent on the insulin action.

**Figure 6:**
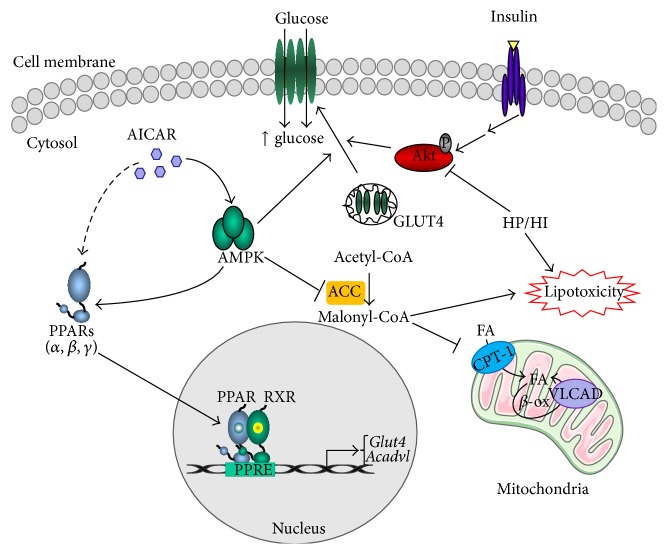
Schematic representation of the potential mechanisms by which AICAR regulates cardiac metabolism and protects HL-1 cardiomyocytes from the HP/HI-induced lipotoxicity and insulin resistance. HP/HI stimulation induces insulin resistance by promoting the intramyocellular lipid build-up, which in turn inhibits the insulin signalling pathway and the AKT-mediated GLUT4 membrane translocation and glucose uptake. AICAR treatment takes part in the regulation of the cardiac metabolic adaptation at several levels. First, AICAR short-term stimulation promotes the inhibition of ACC, thereby reducing the levels of the allosteric inhibitor of CPT-1 malonyl-CoA and regulating the fatty acid mitochondrial *β*-oxidation. In addition, AICAR-induced AMPK activation promotes GLUT4 membrane translocation through non-insulin dependent mechanisms. Finally, AICAR stimulation induces the three PPARs mRNA levels and controls the expression of some key PPAR-target genes, such as* Acadvl* and* Glut4*, involved in both glucose and fatty acid cardiac metabolism. Therefore, through these different mechanisms, AICAR is able to regulate both the acute metabolic response and the long-term metabolic adaptation in cardiac cells.

Although further studies are necessary to fully clarify the role of AMPK-PPAR axis in the metabolic cardiac adaptation, here we show that the AMPK activator AICAR is able to protect cardiac HL-1 cells from the HP/HI-induced intramyocellular lipid accumulation and insulin resistance at several levels. First, after AICAR short-term exposure, AMPK acts as kinase regulating by phosphorylation key enzymes involved in the acute cellular response to counter energy deficiency. In addition, AMPK is also able to regulate metabolic adaptation in response to sustained AICAR stimulation through transcriptional control of genes involved in the metabolic response, such as the PPAR-target genes* Acadvl* and* Glut4* (Figure 6).
